# Editorial: Emerging topics in human physiology

**DOI:** 10.3389/fphys.2025.1594305

**Published:** 2025-03-25

**Authors:** Joaquín García-Estañ López, Ginés Viscor, Luis Monteiro Rodrigues

**Affiliations:** ^1^ Centro de Estudios en Educación Médica, School of Medicine, University Murcia, Murcia, Spain; ^2^ Cell Biology, Physiology and Immunology Dep., School of Biology, University Barcelona, Barcelona, Spain; ^3^ School of Health Sciences, Universidade Lusófona, Lisboa, Portugal

**Keywords:** human physiology, special issue, emerging topics, big data, cutting edge research

The present Frontiers Emerging Topic in Human Physiology was launched by the end of 2022 with the 2nd International Meeting of the Portuguese Physiological Society marking a significant moment in the post-pandemic landscape. This endeavor underscored the importance of preserving and disseminating research and knowledge as we navigate such troubled waters, facing unprecedented challenges, highlighting our resilience and adaptability in the face of adversity. By fostering collaboration and innovation, this Research Topic addressed emerging health issues and promoted cutting-edge research. More important, it served as a platform for physiologists to share insights, drive discussions, and inspire new ideas, ultimately contributing to the advancement of physiology and related fields.

Eight groundbreaking articles have been compiled, demonstrating that Physiology is thriving. Together, these studies enhance our understanding of human physiological adaptation across clinical, athletic, and technological spheres. Prominent themes include personalized medicine, adaptive training protocols, and innovations in diagnostics. Notably, the integration of wearable technologies with physiological insights holds significant promise for advancing clinical management and optimizing performance.

The first article (Kochanowicz et al.) discusses the acute inflammatory response in elite gymnasts by comparing inflammatory responses in elite male gymnasts (EAG) versus physically active men (PAM) after upper- and lower-body Wingate anaerobic tests. The key findings were that EAG showed superior performance in upper-body power output, and that upper-body exercise caused greater IL-6 increases in EAG, while lower-body exercise induced higher IL-6 in PAM. Finally, the anti-inflammatory IL-10 levels remained elevated in EAG post-exercise, correlating with baseline iron status in PAM. The results suggest that gymnastic training modulates iron-dependent anti-inflammatory responses during intense exercise.

The second article in this series (McLaughlin et al.) deals with caffeine bioavailability during exercise. Researchers examined how exercise-induced physiological changes affect caffeine’s unbound fraction (*f*_u_) and found that high-intensity cycling caused significant core temperature rise (+1.37°C) and blood acidification (pH −0.12), but neither *f*_u_ (0.86 → 0.75) nor paraxanthine (0.59 → 0.70) changed significantly and the metabolic ratio ([paraxanthine]/[caffeine]) remained stable under exercise conditions. The results indicate that the pharmacological activity of caffeine is not substantially altered by acute exercise stressors.

In the third article by Liu et al., renal pelvis pressure dynamics was studied in a murine model in order to identify factors influencing renal pressure measurements. The main findings were that perfusion rates (15–120 mL/h) directly correlated with pelvic pressure, the ureter obstruction sites: ureteropelvic junction (UPJ) vs. ureterovesical junction (UVJ) caused differential pressure increases and the bladder emptying protocols significantly affected pressure readings. A good precision regression model is proposed to provide a reference for evaluating renal pelvis pressure. These findings have important implications for obstructive uropathy diagnosis.

In an interesting paper, performed on elite divers (Kjeld et al.), hemoglobin concentration changes and blood shifts during dry static apnea in elite breath-hold divers were observed revealing unique physiological adaptations. Thus, 162% greater blood shift from extremities than from spleen during apnea, elevated hemoglobin concentrations compared to controls (p < 0.05) and no significant left ventricular myocardial mass changes during 4-min apneas, which are interesting responses since these findings demonstrate that blood shift is not towards the heart during dry apnea in humans.

Advanced physiological data acquisition was also applied to through wearable devices for seizure monitoring in the PreEpiSeizures project (Abreu et al.). The authors achieved 2,721 h of multimodal biosensor data from 59 epilepsy patients, matching ECG, high-quality video and EEG systems which allow the identification of 18 technical challenges in wearable epilepsy monitoring in home and clinical settings. The authors state, and we agree, that this work establishes a new and promising framework for daily life seizure detection technologies.

Leite-Moreira et al. studied myocardial compliance in *ex vivo* rabbit right ventricular papillary muscles under ischemic and non-ischemic conditions. Pressure-volume hemodynamics was also evaluated in an experimental *in vivo* acute myocardial infarction (MI) induced by left anterior descending artery ligation in rats. Results showed that ischemia abolishes stretch-induced myocardial compliance and that PKG activation restored 68% of compliance in ischemic tissue. Interestingly, sildenafil-enhanced compliance improved cardiac output by 22% post-MI. These findings suggest that PDE5 inhibitors could optimize volume management in acute MI, An interesting work by Monteiro Rodrigues et al. reported the role of central control of microcirculation through cutaneous perfusion changes induced by two different maneuvers by simultaneous monitoring of both feet by laser Doppler flowmetry and photoplethysmography. They demonstrated systemic hemodynamic responses to local stimuli (venoarteriolar reflex and reactive hyperemia), and blood redistribution between vascular plexuses via central mechanisms while showing age-independent response patterns across cohorts previously studied. The authors state that their findings challenge traditional local reflex models of microcirculatory regulation, highlighting the role of a centrally mediated reflex, documented in real-time through functional imaging, and with pending identification of its sensors and efectors.

Finally, an opinion piece by Li et al. proposed an automatic heart rate clamp, a practical tool to control internal and external training loads during aerobic exercise. They compared automatic versus manual control methods to maintain a target HR by monitoring internal/external load proportionality thus allowing real-time adjustments for cardiovascular drift compensation. AutoHR clamp method allows more precise control of the HR response compared to manual adjustment of external load. However, as the study was a pilot test with only five participants, further investigations are required to confirm these results. The authors conclude that the internal load of exercise can be easily controlled through the adjustment of external workload via an automatic HR clamp. Such a method allows for relative exercise intensity to be maintained constant both within and between exercise sessions, notably when training with additional environmental stress.

In conclusion, this Research Topic contributes to underscore the evolving significance of physiology at present. Clearly, the growing utility of wearable devices and advanced big data collection methods suggest new understandings and research directions within modern human physiology. In any case, this deliverable, only possible by this combined initiative and effort from Topic Editors, emphasizes the crucial role of researchers in the dissemination of knowledge. Such efforts are essential to foster a healthier and more informed society as we progress together into the future, paving the way for improved health outcomes and a deeper comprehension of the physiological factors that influence our daily lives.

